# Cognitive Factories: Modeling Situated Entropy in Physical Work Carried Out by Humans and Robots

**DOI:** 10.3390/e20090659

**Published:** 2018-09-01

**Authors:** Stephen Fox, Adrian Kotelba, Ilkka Niskanen

**Affiliations:** VTT Technical Research Centre of Finland, FI-02044 VTT, Finland

**Keywords:** artificial intelligence, cognitive load, embodied cognition, entropy, factory, pragmatics, Information Theory, robotics, situated cognition

## Abstract

Entropy in factories is situated. For example, there can be numerous different ways of picking, orientating, and placing physical components during assembly work. Physical components can be redesigned to increase the Information Gain they provide and so reduce situated entropy in assembly work. Also, situated entropy is affected by the extent of knowledge of those doing the work. For example, work can be done by knowledgeable experts or by beginners who lack knowledge about physical components, etc. The number of different ways that work can be done and the knowledge of the worker combine to affect cognitive load. Thus, situated entropy in factories relates to situated cognition within which knowledge is bound to physical contexts and knowing is inseparable from doing. In this paper, six contributions are provided for modelling situated entropy in factories. First, theoretical frameworks are brought together to provide a conceptual framework for modelling. Second, the conceptual framework is related to physical production using practical examples. Third, Information Theory mathematics is applied to the examples and a preliminary methodology in presented for modelling in practice. Fourth, physical artefacts in factory production are reframed as carriers of Information Gain and situated entropy, which may or may not combine as Net Information Gain. Fifth, situated entropy is related to different types of cognitive factories that involve different levels of uncertainty in production operations. Sixth, the need to measure Net Information Gain in the introduction of new technologies for embodied and extended cognition is discussed in relation to a taxonomy for distributed cognition situated in factory production. Overall, modelling of situated entropy is introduced as an opportunity for improving the planning and control of factories that deploy human cognition and cognitive technologies including assembly robotics.

## 1. Introduction

Factories that deploy artificial intelligence (AI) can be described as cognitive factories. The deployment of AI in factories is argued to be necessary to bring the speed and consistency of fully automated mass production to the manufacturing of individual products [1,2]. By 2018, cognitive factories will have existed as a manufacturing systems goal for more than 10 years, yet manufacturers seeking to increase the individuality of products, such as Mercedes and Toyota, are reducing the number of assembly robots and increasing the number of human operatives. This is because robots are not able to match human embodied cognition in acting competently under uncertainty. Importantly for the production of individual goods, the embodied cognition of human operatives continues to provide unsurpassed flexibility for diverse sensorimotor/psychomotor tasks in assembly work [3,4]. Reliance on human workers for sensorimotor/psychomotor tasks in the production of individual goods continues in spite of many efforts to improve the embodied cognition of robots [5,6]. Nonetheless, human operatives make many errors during production work [7,8].

One step towards improving the performance of both assembly robots and human operatives is modelling situated entropy. For example, situated entropy can provide a measurement of task uncertainty that is applicable to both assembly robots and human operatives within computational conceptualizations of embodied cognition [9,10]. At the same time, measurement of task uncertainty in terms of situated entropy can provide targets for Information Gain [11,12], which can be worked towards through application of, for example, industrial engineering methods and situation awareness modelling [13]. The remainder of the paper comprises six sections. In Section 2, background is provided about the modelling of situated entropy for physical work. Then, in Section 3, conceptual framework for modelling is introduced. Subsequently, in Section 4, results from modelling are presented. In Section 5, following from the example results, a preliminary modelling methodology is presented. In the penultimate Section 6, implications for the production of individual goods are discussed from the perspectives of theory building, applied research, and practice. In the concluding Section 7, principal contributions to manufacturing systems knowledge are described.

## 2. Background

Entropy is larger when there is a larger number of ways in which something can happen. For example, there is little uncertainty about flipping a fair coin: the outcome will be heads or tails with equal probability. This uncertainty can be quantified as an entropy of 1.00. By contrast, there is more uncertainty about rolling a fair six-sided die. This is because there are more ways in which something can happen as the fair coin can land on two faces but the fair die can land on six faces. This increased uncertainty can be described as an entropy of 2.58 [14]. In physical work, if a component can be fitted in six different ways with equal probability (i.e., entropy 2.58) and it is redesigned so it can be fitted in only two ways (i.e., entropy 1.00) there is an Information Gain of 1.58. Moreover, if a component is redesigned so that it can be fitted in only one way (i.e., entropy 0.00), there is an Information Gain of 2.58. Similarly, uncertainty arising from manufacturing instructions can be reduced through information and communication design that reduces sources of ambiguity [15].

Higher Information Gain per knowledge unit has been described as increased knowledge helpfulness to knowledge users [16]. Important knowledge users in assembly of individual products on factory floors include assembly robots and human operatives. It is essential that increased Information Gain reduces their robot computational loads and human cognitive loads. This is because there are high computational/cognitive loads involved in sensorimotor/psychomotor assembly work, which can be increased to overload by uncertainties in the assembly of individual goods [3,17]. In particular, even low-level sensorimotor robot work can involve more computational load than the high-level reasoning at which AI excels, for example when beating human world champions at board games [18,19]. Moreover, transfer of sensorimotor skills by robots to new settings can involve very high computation loads [20,21,22]. For human operatives, there can be high cognitive loads during the practice and feedback required to develop embodied neurological templates for psychomotor skills [23]. Subsequently, embodied neurological templates are flexible enough to enable human experts to transfer their psychomotor skills to new settings [24]. However, the performance of human experts can deteriorate if knowledge units involve high cognitive loads: i.e., overload leads to underperformance [25,26,27].

Accordingly, Information Gain and computational/cognitive overload need to be addressed together in modelling. However, in doing so, it is important to take into account factors that affect robot workers and human operatives differently. For example, more colorful presentation of information may only increase computational load for assembly robots. By contrast, more colorful presentation of information may increase human cognitive absorption and facilitate Information Gain for human workers [28].

## 3. Conceptual Framework

In this section, findings are described from review of extant theoretical frameworks. Then, the conceptual framework for modelling introduced.

### 3.1. Extant Theoretical Frameworks

As explained in the following paragraphs, the modelling reported in this paper is informed by two apposite theoretical frameworks. The first theoretical framework is provided by Situated Cognition, Embodied Cognition, and Embodied Cognitive Load Theory [29,30,31,32]. The second theoretical framework is provided by Pragmatics of Material Interaction, Performative Pragmatics and Relevance Theory [33,34,35,36].

Embodied Cognition describes the body beyond the brain taking physically constitutive roles in cognitive processing [37,38,39]. Embodied Cognition relates to broader Situated Cognition, within which knowledge is bound to physical contexts and knowing is inseparable from doing. Situated Cognition and Embodied Cognition are relevant to artificial intelligence (AI) as well as human intelligence. For example, Behaviour-based Robots develop embodied knowledge through trial-and-error sensing and reacting to situations. This situated development of embodied AI offers opportunities to overcome the barrier to scaling up robotics that would otherwise be caused by programmers having to anticipate every situation and pre-program every response in advance. However, the computational demands of embodied AI can easily escalate, for example because of complexity in robot vision data processing [40,41,42]. Embodied Cognitive Load Theory (ECLT) can be applied to structure Information Gain, and at the same time structure prevention of cognitive overload/computational overload that leads to underperformance. Within ECLT, there are three types of cognitive load: intrinsic, extraneous and germane. Intrinsic cognitive load can be related to fundamental characteristics of a task. For example, assembling a vehicle engine with 50 plus parts involves more intrinsic cognitive load than assembling a three-part toy engine. Extraneous cognitive load can be related to cognitive load involved in identifying the correct assembly tools for an assembly operation. Germane cognitive load can be related to the work put into creating a permanent store/schema of assembly work knowledge [30,43].

Increasing Information Gain while reducing robot computational load and human cognitive load, can be structured further through reference to three theoretical formulations of work pragmatics: Pragmatics of Material Interaction, Performative Pragmatics, and Relevance Theory. Within the Pragmatics of Material Interaction, assembly workers interact with materials, such as physical components, which are designed to be interacted with in particular ways by the assembly worker in order to fulfil a productive intention, such as the efficient assembly of a physical product [34]. Within Performative Pragmatics, information contributes to performance of action, in other words information is performative only if it is issued in the course of the doing of an action [33]. Performative information [44] can be commissive (e.g., promise to take action); directive (e.g., commands/requests), declarations (e.g., rejections/approvals), and/or expressives (e.g., congratulations/thanks). Within Relevance Theory, the probability of information being acted upon increases with the probability that it connects with other available sources to yield a positive cognitive effect, such as settling a doubt, correcting a mistaken impression, answering a question, and/or improving knowledge on a certain topic. Thus, information that is intended to performative either indirectly through component design or directly through commands etc. needs to connect with other available sources, such as extant cognitive/computational schema, in order to be easily processed: i.e., understood with minimal cognitive/computational effort [36]. A summary of the extant theoretical frameworks is provided in Table 1. In the following paragraphs these are integrated to provide a conceptual framework for modelling.

### 3.2. Conceptual Framework for Modelling

Design engineers can design Information Gain into assembly operations and, at the same time, design cognitive/computational load out of assembly operations, through at least three engineering design strategies that can be derived from combining extant theoretical frameworks described above.

First, Information Gain can be increased by improving Pragmatics of Material Interaction during assembly operations. For example, physical components can be designed to be symmetrical or obviously asymmetrical. Such component design makes it immediately apparent how a component should be picked/placed, and so reduces intrinsic cognitive load while increasing Information Gain [45,46]. Furthermore, the Information Gain provided by the physical component is much higher than the opposite scenario where a physical component that has no immediately obvious orientation for picking and placing. This example illustrates one opportunity for reduced intrinsic embodied cognitive load to facilitate Information Gain. Also, this example illustrates that designing for reduced embodied cognitive load can reduce entropy: i.e., reduce the amount of information needed to represent an event, in this case, the event of component picking/placing.

Second, Information Gain can be increased by improving Performative Pragmatics during assembly operations. For example, directive information provided by visual control boards for assembly tools can be designed to increase Information Gain by maximizing comprehension and engagement. At the same time, design of visual control boards can minimize vagueness and ambiguity, and so reduce extraneous cognitive load. Such design of visual control symbols/templates makes it immediately apparent what assembly tools should be used for what assembly tasks [47,48]. Furthermore, the Information Gain provided by the symbols/templates is much higher than the opposite scenario where a method of assembly tool control that is not intuitively understandable. This example illustrates one opportunity for increasing Information Gain, and reducing extraneous embodied cognitive load. Again, this example illustrates that designing for increased Information Gain and reduced embodied cognitive load can reduce entropy: i.e., reduce the amount of information needed to represent an event, in this case, the event of identification of correct tools for assembly operations.

Third, in accordance with Relevance Theory, Information Gain can be increased by designing to maximize the connection of knowledge units, such as physical component, visual control boards, etc., to extant computational/cognitive schema: i.e., through design to maximize relevance. Such design increases the probability of information being acted upon as it increases the probability that it yields a positive cognitive effect, such as improving knowledge. This emphasis on schema extends design work to systems engineering, for example, designing factory operations to have assembly workstations that provide consistent layouts but with sufficient flexibility to enable efficient assembly of individual goods. This can increase Information Gain and, at the same time, reduce germane cognitive load involved in developing computational/cognitive schema of assembly work knowledge [49,50]. Furthermore, the Information Gain provided by designing for relevance is much higher than the opposite scenario where an overall production process accumulates ad hoc in response to production crises etc. This example illustrates one opportunity for reduced embodied germane cognitive load to facilitate increased Information Gain. Again, this example illustrates that designing for reduced embodied cognitive load can reduce entropy: i.e., reduce the amount of information needed to represent an event, in this case, an assembly process.

As illustrated by these examples, knowledge units for Information Gain extend beyond written information to physical components, visual control boards, and other physical artefacts used in the assembly of individual physical products. A summary of the conceptual framework for modelling is provided in Table 2.

## 4. Modelling Examples

### 4.1. Discrete Equal Probability

Application of the conceptual framework for modelling entropy in order to inform cognitive load reduction and Information Gain is described in the following paragraphs. Entropy can be given precise mathematical definition. Specifically, the entropy (*H*) of a random variable *X* can be written as Equation (1) if the random variable *X* takes on values in a set *X* = {*x*1, *x*2, … *x*n} and is defined by a discrete probability distribution *P*(*X*):

(1) $$H\left(X\right)\;=-\underset{x\epsilon X}{\sum }P\left(x\right)\log P\left(x\right)$$

Entropy is high when uncertainty about an event is high. For example, as shown by Equation (2), there is entropy of 2.58 if a component can be orientated in six different ways with equal probability. Then, entropy reduces to 0.00 if the component is redesigned so it can be orientated in only one way. Hence, redesign brings an Information Gain of 2.58:

(2) $$H\left(O\right)\;=-\underset{o\epsilon \left\{1,2,3,4,5,6\right\}}{\sum }P\left(x\right)\log P\left(x\right)$$

= − [1/6log1/6+1/6log1/6+1/6log1/6+1/6log1/6+1/6log1/6+1/6log1/6]

= 2.584962500721156

As summarized in Table 3, modelling can include measurement of intrinsic, extraneous and germane entropy. Sources of entropy related to physical components include picking, orientating and placing; sources of entropy related to visual control boards include conceptual, presentational and linguistic; sources of entropy related to assembly workstations include component access, work sequence and physical positioning.

A summary of modelling Information Gain targets for one assembly task at one workstation is shown in Table 4. This shows that the design of the physical component allows it to be picked in one way, orientated in three ways, and placed in two ways, which leads to an Information Gain target for that physical component in this task of 2.58. The design of the visual control board for the tool needed in placing the physical component has no conceptual ambiguity. This is because the full outline shape of the tool is inset into visual control board. Thus, there is direct conceptual equivalence between the image on the visual control board and the physical tool itself. Also, there is no linguistic ambiguity because words are not used on the visual control board. However, the outline shape of the tool to be used is positioned too close to the outline shapes of two similar sizes of the same tool type. Hence, it can be difficult to differentiate between the three, which leads to an Information Gain target for the visual control board in this task of 1.58.

The design of the assembly workstation allows two different ways of accessing the physical component from its storage bin. Also, there are two ways of sequencing the work with the physical component, and five different physical positions with which the work with the physical component can be done. This leads to an Information Gain target for the assembly workstation in this task of 4.32.

### 4.2. Discrete Unequal Probability

As summarized in Table 5, targets will differ from those recorded in Table 4 when there is unequal distribution across the different ways of carrying out the work in one task.

These unequal distributions may be unintended. For example, a human expert in the task will usually carry out the orientation in the one way that is most efficient: except for sometimes when lacking motivation due to being bored, etc. [51,52]. This can lead to one way of orientating the physical component being used two out of three times and the other two ways being used equally: leading to a distribution of 2/3, 0.5/3, 0.5/3 rather than 1/3, 1/3, 1/3. This leads to entropy of 1.25 rather than 1.58. By contrast, poor design of the visual control board could lead to there being an equal distribution of recurring uncertainty and hence the same target of 1.58 as shown in Table 4. Yet, as shown in Equation (3), the entropy of physical positioning (*PP*) may be much less if one way of physical positioning is taken four times out of five, but not every time due factors such as loss of motivation due to boredom, etc., [50,51,52]. This can lead to an entropy and Information Gain target very similar to when there are two ways of doing the same task. In particular, the Information Gain target is 1.0006, which is very close to 1.0 and close to 1.32 less than entropy of 2.32 when each of the five ways is used with equal frequency:

(3) $$H\left(PP\right)=-\underset{pp\epsilon \left\{1,2,3,4,5\right\}}{\sum }P\left(x\right)\log P\left(x\right)$$

= − [4/5log4/5 + 0.2/5log0.2/5 + 0.2/5log0.2/5 + 0.2/5log0.2/5 + 0.2/5log0.2/5]

= 1.0005594662738457

### 4.3. Joint Entropy

By contrast, sources of entropy can be reduced for both human operatives and assembly robots by designing individual products to be based on modular product architectures and families of components [53,54]. Such engineering design can increase the number of times that the same knowledge units, such as physical components, are used in different knowledge categories, such as products. This can enable human operatives to become increasingly familiar with the existence of knowledge units and the understanding of their meaning. Also, assembly robots can carry out the same task repeatedly in slightly different settings and potentially learn how to carry out the task with increasing efficiency [55]. Entropy arising from repeated use of the same task in different products can be modelled in terms of joint entropy. For example, familiarity (familiar not familiar) with product variation (*F*) and ways of orientating (3/5, 1/5, 1/5) physical component (O) can be modelled in terms of joint entropy as shown by Equation (4):

(4) $$H\left(F,O\right)\equiv H\left(P\left(F,O\right)\right)=-\underset{f\in F}{\sum }\underset{o\in O}{\sum }P\left(f,o\right)\log P\left(f,o\right)$$

In this case, joint entropy involves the following joint distributions:

*P*(*familiar, 3/5 orientation*) = *2.5/5* (5)

*P*(*not familiar, 3/5 orientation*) = *0.5/5* (6)

*P*(*familiar, 1/5 orientation*) = *0.75/5* (7)

*P*(*not familiar, 1/5 orientation*) = *0.25/5* (8)

*P*(*familiar,1/5 orientation*) = *0.75/5* (9)

*P*(*not familiar, 1/5 orientation*) = *0.25/5* (10)

Applying Equation (8) as show below entropy is 2.08:

*H(F,O)* = − [2.5/5log2.5/5 + 0.5/5log0.5/5 + 0.75/5log0.75/5 + 0.25/5log0.25/5 + 0.25/5log0.25/5 + 0.25/5log0.25/5](11)

*H(F,O)* = 2.0854752972273345 (12)

### 4.4. Conditional Entropy

Conditional entropy involves the entropy of one knowledge unit being dependent upon another. For example, without engineering design of product architectures and families of components for simplicity of assembly, it could be possible for knowledge of component orientation *(O)* to be dependent upon familiarity *(F)* with product type. Conditional entropy can be written as shown in Equation (13) when the vertical bar | means “given that”:

*H(O*|*F)* (13)

If *O* is completely dependent upon *F*, then *O* has 0.00 entropy when there is no uncertainty of *F*. Accordingly, the relationship between conditional entropy and joint entropy can be expressed as shown in Equation (14), where full knowledge of *F* results in *O* no longer being conditional on *F*:

*H (O|F) + H(F) = H(F,O)* (14)

As shown in Equation (15), conditional entropy can be described in very similar terms to joint entropy as shown in Equation (4):

(15) $$H\left(O|F\right)=-\underset{f\in F}{\sum }\underset{o\in O}{\sum }P\left(f,o\right)\log P\left(o|f\right)$$

However, rather than calculate conditional entropy, it is preferable to make the its elimination a priority in the engineering design of assembly work. This is important to avoid chains of uncertainty spreading throughout factory operations, which can lead to multiple problems for productivity and quality. Conditional entropy can be reduced by replacing iterative processes of action-observation-action with processes where repeated observation to determine next actions is not needed. For example, car bodies can be fabricated by humans who are skilled in the craft of panel beating. This involves sequential action and observation as the skilled human operative starts to beat sheet metal into compound curves with, for example, pear shaped mallets. Then, observes the emerging panel shape to determine where next to beat the sheet metal with what force using which mallet. This sequential process is used in some low-volume sports car production when relatively easily formed aluminum alloy is used. However, panel beating has been replaced in the vast majority of car production with automated presses that produce body panels in a few seconds. Similarly, but with more versatility, additive manufacturing such as 3D printing can replace time-consuming cost-generating iterations of craft-based action and observation with a continuous automated process [56].

### 4.5. Differential Entropy

It is not always realistic to model entropy as having discrete probabilities. For example, beginners are likely to try out more ways of performing tasks than experts [57,58]. However, there is uncertainty about how many ways they will try out for each task. Hence, it is appropriate to apply continuous probability distributions such as the Normal Distribution. Differential entropy, which is the entropy of a continuous random variable, can be described as shown in Equation (16) where natural logarithm (ln) is applied to square root of pi times the variance (*σ^2^*). Here, variance is the principal determinant of differential entropy as the other factors are constant:

(16) $$h(x)\;=\;½+\ln(\text{√}2\pi \sigma^{2})$$

For example, the mean of ways beginners try out for each task can be 3.0 and the variance can be 2.0. In that case, the differential entropy can be calculated as shown in Equation (23). The multiplication by 1.442695041 in Equation (22) is applied to enable differential entropy to be expressed in the same unit of measurement as discrete probability distributions for entropy. This is useful, for example, to enable comparisons to be made [59]:

2 × π = 6.28318530718(17)

6.28318530718 × 2.00 = 12.5663706144(18)

√12.5663706144 = 3.5449077018(19)

ln 3.54490770182 = 1.2655121235(20)

½ + 1.2655121235 = 1.7655121235(21)

1.7655121235 × 1.442695041= 2.54709558540(22)

*h(X)* = 2.54709558540 (23)

## 5. Preliminary Modelling Methodology

In accordance with the conceptual framework encompassing cognitive load and work pragmatics, the examples above illustrate that physical artefacts of production, such as components, control boards and workstations can carry situated entropy and can bring Information Gain. Based on this conceptualization of physical artefacts as pragmatic knowledge units in cognitive factories, a preliminary methodology for modelling situated entropy and its reduction through Information Gain is introduced in this section. In particular, the focus of the preliminary methodology is *Net* Information Gain brought about by six strategies, all of which can be applied in reducing different types of situated entropy.

### 5.1. Methodology Focus—Net Information Gain

Adaptations of knowledge units or the introduction of new technologies to supplement knowledge units can introduce additional sources of entropy. For example, augmented reality (AR) work instructions overlay computer-generated information on the real world environment; often using semi-transparent head-mounted displays. Since the new Millennium, it has been argued that AR can provide a better medium for assembly information than traditional media [60]. However, as summarized in Table 6 and Table 7 AR introduces new sources of entropy from uncertainties related to the fit of AR technology to task, place and person. For example, the information communicated through AR must be the up-to-date digital information for the exact features of the particular task for the specific product type. This can involve complicated data management challenges when there are an increasing number of new options for product types. Also, the workplace must be suitable for enabling reliable wireless transmission of large data files. In addition, assembly operatives who use AR must have natural vision capable with dealing with AR displays without suffering headaches, etc.

Hence, even if, as shown in Table 7, AR reduces entropy from presentational ambiguity to zero, AR can introduce entropy into the communication of the information [15,61]. Thus, rather than Net Information Gain, there can be net increase in situated entropy. Let X denote state of product after assembly, Z denote information contained in augmented reality work instructions which human operator understands as Y due to possible misfit to the task or communication errors. Accordingly, as summarized in Equation (24)–(26) the modelling of situated entropy should take into account that steps taken to reduce extant situated entropy can introduce new sources of situated entropy and confound Information Gain:

H(X,Y|Z)(24)

= H(X|Z)(25)

+ H(Y|X,Z)(26)

Equations (25) and (26) follow from Equation (24) by the chain rule for conditional entropy. Conditional entropy H(X|Z) in Equation (25) is commonly referred to as equivocation and corresponds to the uncertainty in the product assembly from the work instruction point of view. A case that is of specific interest is when H(X|Z) = 0. By its definition H(X|Z) = 0 when X becomes deterministic after observing Z. In other words, the assembly of the product is deterministic given work instructions as shown in Table 7. On the other hand, conditional entropy H(Y|X,Z) in Equation (26) can be referred to as prevarication as it represents the uncertainty in the operator’s understanding of work instructions from the technical writer point of view.

### 5.2. Methodology Strategy One: Apply Engineering Methods

The examples in Section 4 illustrate how situated entropy can be reduced through application of engineering methods that increases Information Gain intrinsic to physical components. The reengineering of production processes that include arcane craft practices can involve introduction of automated processes that can reduce conditional entropy. The reengineering of physical components to reduce the number of ways they can be picked, orientated and placed can reduce discrete entropy. The reengineering of product types can lead to rationalization involving introduction of product architectures and families of parts that can reduce joint entropy. Thus, as summarized in Equations (27) to (29) the application of engineering methods can reduce situated entropy from the complexity of conditional entropy to the comparative simplicity of discrete entropy with few ways of carrying out a task. Typically, the lower the situated entropy, the higher the potential for automation. However, often successful application of engineering methods involves large-scale capital investment. For example, replacing the complex conditional entropy of panel beating with the minimal discrete entropy of automated presses can involve capital investments, which are so large that tens of thousands of the same shape panel have to be sold. The more variation in customer requirements there are, the less potential there can be for entropy reduction through engineering methods alone [3]. Let random variables X, Y, and Z denote the possible state of physical components in the final product, for example, location and orientation in three-dimensional space. Furthermore, let us assume that random variables X, Y, Z take values from sets A, B, and C with sizes |A|, |B|, and |C|, respectively. Then, by properties of joint entropy we obtain with equality in Equation (28) if and only if X, Y, and Z are independent and equality in Equation (29) if and only if X, Y, Z are uniformly distributed on A, B, and C, respectively. In words, the application of engineering methods that reduce the number of ways of carrying out a given task leads to reduction of upper bound on joint entropy as shown in Equation (29). The upper bound Equation (28), on the other hand, represents the worst-case scenario where components x, y, and z are independently designed. If they are jointly designed, for example, the location and orientation of x determines the location and orientation of y and location and orientation of y determines in turn the location and orientation of z, then H(Y|X) and H(Z|X,Y) are zero in Equation (27) which leads to significant reduction in situated entropy. Similar conclusions can be drawn when x, y, and z represent product architectures, families of products, or modules rather than physical components:

H(X,Y,Z) = H(X) + H(Y|X) + H(Z|X,Y)(27)

<= H(X) + H(Y) + H(Z)(28)

<= log(|A|) + log(|B|) + log(|C|)(29)

### 5.3. Methodology Strategy Two—Introducing Embedded Artificial Cognition

A complementary strategy can be to deploy embedded artificial cognition through advanced automation technologies such as self-adapting production lines [62]. Such production technologies can reduce the amount of knowledge needed by workers in low volume manufacturing of a mix of products. Detailed description of such technologies is outside the scope of this paper. However, it is important to note that the potential of such technologies to reduce situated entropy is accompanied by their potential to introduce new sources of germane entropy: for example, through disruption of established workstation layouts. In particular, introduction of embedded technologies can reduce the number of iterations of traditional actions and observations, and so reduce traditional sources of conditional entropy. Nonetheless, as summarized in Equation (30) there can be new sources of conditional entropy. For example, expert panel beaters make many observations as they look at the emerging shapes of sheet metal that they are working with mallets etc. By contrast, the objectives of deploying a self-adapting production line can include eliminating the need for human observations. However, this type of “black box” can lead to outcomes without explanation. Such opacity of artificial cognition in workplaces is being addressed by research into improving its transparency [63]. Meanwhile, the operation of artificial cognition needs to involve human observation of control panels/dashboards etc., which results in traditional observations such as those of panel beaters being replaced by new types of observations. Let Xi denote the action of self-adapting line applied to the ith component of a product and Yi denote the observation of the outcome of the corresponding action. For simplicity, let us consider only two physical components. Then, by the lower-additivity property of conditional entropy we obtain:

H(X1,X2|Y1,Y2) <= H(X1|Y1) + H(X2|Y2)(30)

with equality if and only if (X1|Y1) and (X2|Y2) are stochastically independent. In words, performing action on many components and jointly observing its outcome at once rather than in sequential way reduces the situated entropy. However, the observations needs to be done jointly (Y1,Y2) which, in turn, implies that information presented to possible human supervisor is of new type. In particular, several observations are first combined and then presented to an observer.

### 5.4. Methodology Strategy Three—Introducing Enactive Artificial Cognition

A further complementary strategy can be to deploy enactive artificial cognition through new technologies. For example, electrochromic materials that change their colour or opacity due to actions such as the application of a voltage; thermochromic materials that change in colour depending on their temperature; and photochromic materials that change colour in response to light. More sophisticated is adaptive structure technology, i.e., adaptatronics, which combines conventional fabricated structures with active material systems that include sensor and actuator functioning. In connection with suitable adaptive controller systems, adaptive structure systems can adapt to their respective operational environment [64]. Exactly how such technological advances, which introduce some rudimentary possibilities for enactive artificial cognition, can be applied in the production of physical goods is an open question outside of the scope of this paper. However, it is apparent that colour changes in smart materials can introduce new sources of extraneous cognitive load alongside potential Information Gain. Hence, colour changes need to be related clearly to explanation of what the colours means in the context in which they are displayed. Equations (24)–(26) in Section 5.1. are relevant to calculation of Net Information Gain from such sources.

### 5.5. Methodology Strategy Four—Enhance Embodied Artificial Cognition

In addition to seeking to introduce Net Information Gain through engineering methods, embedded artificial cognition and enactive artificial cognition, steps can be taken to reduce situated entropy by enhancing the embodied cognition of those doing the work. The acquisition robot psychomotor skills can involve the “imitation learning” of “robot apprenticeship learning” and “robot learning from demonstration”. Both involve demonstration, observation, imitation, practice, and feedback [65]. A proposed advantage of robot learning from demonstration is that it can enable adaptation of skills to new settings with minimal extra programming. Indeed, a goal of robot learning from demonstration is that humans can teach robots new tasks without those humans having any knowledge of programming [66]. This can involve application of advances in computer vision concerned with human activity recognition [67]. At the same time, it is important to determine which aspects of demonstrations are essential to achieving the desired outcome, through data analyses and/or from advice by human experts. Also, it is necessary to determine how demonstrations can be imitated by robots, which perceive differently to humans and are physically different to humans [68]. Furthermore, as robots do not naturally position themselves in the same way humans for optimal performance, combined motion and task planning is needed for robot workers [69]. Given the challenges of robot learning from demonstration, it is important to engineer production tasks for least action. In particular, the Principle of Least Action is founded upon scientific observations that nature tends to act as simply as possible: for example, by taking a path between two points that requires the least action. Indeed, it has been argued that it is “Nature’s Command” to “follow the path of least action” [70]. The Principle of Least Action has been found to be able to simplify explanation of a wide range of highly complex phenomena involving physical motion. However, unlike an equation of motion, Principle of Least Action (PLA) does not explicitly specify what will happen. Rather, PLA asserts that the action will be the least of any conceivable actions with the path of least action offering minimal action compared to all possible paths [71,72]. As summarized in Equations (31) and (32), engineering tasks towards least action can involve going from the conditional entropy of eight sequential steps of position, motion and observation to the joint entropy of four flowing motions such as seamless flow between positioning, picking, orientating and placing. For simplicity, let us consider only two motions of a robot. Let X1 and X2 denote the motions of robot and Y1 and Y2 denote the corresponding observations of robot state. Then, by the lower-additivity property of conditional entropy we obtain:

H(X1,X2|Y1,Y2)(31)

<= H(X1|Y1) + H(X2|Y2)(32)

with equality if and only if (X1|Y1) and (X2|Y2) are independent. In words, it is beneficial to combine a few motions into a one flowing motion because it reduces the situated entropy. Execution of motions as independent motions in a step-by-step way actually leads to the largest possible entropy.

### 5.6. Methodology Strategy Five—Enhance Embodied Cognition of Human Operatives

Also, steps can be taken to reduce situated entropy by enhancing the embodied cognition of human operatives. For example, the embodied cognition of human operatives can be enhanced by wearable robotics such as motorized exoskeletons [73]. However, use of wearable robotics can require additional conscious thought, which impairs smooth performance of psychomotor skills. In particular, getting into the zone of smooth performance of psychomotor skills can be hindered by difficulties of achieving sensory fusion between the human and the wearable robotics. One barrier to doing so is that physical movement in the performance of a psychomotor skill can begin before related brain waves start. Hence, controlling wearable robotics through via brain-machine interface systems (BMI) is an intractable challenge [74]. Improving the ergonomics of wearable robotics could reduce the amount of conscious thought needed in their use, and so increase potential for smooth performance of psychomotor skills [75]. Then, over the longer term, if an individual uses a wearable robotics for thousands of hours, it may be possible that neural plasticity could lead to adaptation of that individual’s relevant brain functioning. In doing so, neural plasticity could facilitate smooth performance of psychomotor skills with the wearable robot. Examples of such effects from neural plasticity are provided by people who develop autonomous understanding of inputs received through Cochlear Implants [76]. However, use of wearable robotics can involve many areas of the brain, and so neural adaptation may take longer or may not be possible. Hence, wearable robotics can introduce new sources of unpredictability in human performance as human operatives have to think about how to move with the exoskeleton instead of just moving naturally. This unpredictability can be modelled in terms of discrete unequal probability for isolated tasks. Modelling in terms of differential entropy for multiple tasks across factory operations can be carried out through use of Equation (20) shown in Section 4.5. Differential entropy is dependent upon variance with increases in variances bringing increasing in differential entropy. Accordingly, variance in the performance of human operatives arising from use wearable robotics is the key consideration in modelling associated differential entropy. Further specificity may be introduced into modelling by taking human biocybernetic cycles into consideration, which involves human decision making and physical action deteriorating due to energy depletion, e.g., as time since food intake increases [77]. Thus, differential entropy can be modelled in relation to times of working day, such as the hour leading up to lunch break have higher variance than preceding hours and overtime hours having further increased variance.

### 5.7. Methodology Strategy Six—Balance Deployment of Artificial and Natural Embodied Cognition

Typically, robots are deployed most in make-to-stock (MTS) mass production of standard goods, while deployed least in engineer-to-order (ETO) one-of-a-kind production of unique original goods. In between these two extremes of MTS and ETO is assemble-to-order (ATO) production of mass customized goods. Modelling of situated entropy can be needed frequently in ATO. This is because components, instructions and workstations can have to undergo frequent changes due to changes in the type and number of choices offered to customers in mass customization business models [3]. Modelling of situated entropy can be needed in some aspects of every customer order for ETO production. This is because the many of the sub-assemblies and assemblies of ETO goods are unique to each customer order [56]. As described in Section 5.2, Section 5.3, Section 5.4 and Section 5.5, there are at least four strategies that can be applied to reduce situated cognition in work carried out by robots. When doing so, it is important to recognize the limitations of artificial neural networks and reinforcement learning in dealing with change. In particular, their performance can rely on the number of cases available as training data and to direct automatic labelling [78]. This reliance can be problematic when there is low repetition of cases (ATO) or when each case is somewhat original (ETO). Although it may be possible in the future for virtual simulation methods to generate new labelled data samples from a few real data samples [79], such solutions require capital investment and computational expertise that are beyond the scope of small ETO companies. Also, ATO and ETO can be considered to be sparse reward environments for reinforcement learning [80] when exactly what has to be learnt keeps changing as ATO options change and new ETO customers have individual requirements. Inverse reinforcement learning that extracts a reward function from observed behaviour [81] is also of limited usefulness when behaviour needs to change frequently as ATO options change and new ETO customers come and go. These challenges make facilitating imitation learning through engineering for least action, outlined in Section 5.5, particularly important for improving robot performance in ATO and ETO. Overall, it is important to eliminate conditional entropy that involves repeated iterations of multi-criteria observations in robot work. Where this is not possible to eliminate conditional entropy, it is desirable to avoid the computational load of computer vision in observations. This can be achieved only to some extent through application of other methods of sensing that are computationally lighter [82]. Hence, in deciding what work can best be done by humans and what work can best be done by robots, work involving conditional entropy where visual observation is essential should be done by humans.

### 5.8. Methodology Summary

All of the methodology strategies can be applied when developing new production processes. Individual methodology strategies can be applied separately when seeking to improve existing production. For example, when many engineering methods have already been applied to production processes and new alternatives need to be considered such as embedding artificial cognition. Throughout, extant situated entropy, new Information Gain, new situated entropy, and Net Information Gain should be measured in terms of conditional, joint, differential or discrete entropy as appropriate.

## 6. Discussion

In this section, implications are discussed for theory building, applied research, and production practice.

### 6.1. Implications for Theory Building

The modelling reported above illustrates that entropy in cognitive factories is situated. For example, there can be numerous different ways of picking, orientating, and placing a physical component. To address the consequent entropy, physical components can be redesigned. Similarly, visual control boards and assembly workstations can bring situated entropy, but can be redesigned to increase Information Gain and reduce situated entropy. Also, the situated entropy involved in physical work is affected by the extent of knowledge of those doing the work. For example, work can be done by knowledgeable experts or by beginners who lack knowledge about physical components, visual control boards, and assembly workstations. The number of different ways that work can be done and the knowledge of the worker, about the ways in which can be done, combine in affecting cognitive load. Thus, situated entropy in cognitive factories relates to Situated Cognition within which knowledge is bound to physical contexts and knowing is inseparable from doing [29].

**Figure 1:**
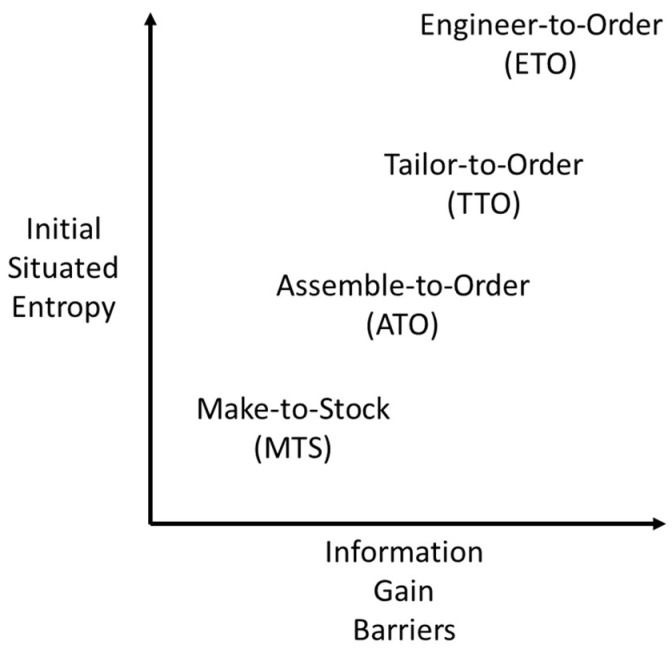
Different levels of situated entropy in physical production.

As summarized in Figure 1, initial situated entropy and barriers to Information Gain can be much lower in Make-to-Stock (MTS) factories than in Engineer-to-Order (ETO) factories. This is because MTS production is characterised by high repetition of standardization in mass production, which is preceded by the rationalization of all components etc., to facilitate full automation involving few, if any, human operatives. By contrast, situated entropy can be much higher in Engineer-to-Order production. This is because ETO goods are intended to be one-of-a-kind in fulfilling the particular requirements of individual customers. Indeed, when variations in Assemble-to-Order (ATO) production are increased towards the level of Tailor-to-Order production (TTO), it can bring about increases in situated entropy that lead to assembly robots being replaced by human operatives [3].

The conceptual framework for modelling summarized in Table 2 incorporates the situated nature of entropy in cognitive factories through inclusion of three types of work pragmatics: Material Interaction Pragmatics, Performative Pragmatics, and Relevance Theory. Overall, pragmatics is concerned with the ways in which context contributes to meaning, and is applied in the development of systems involving the sharing of knowledge among both humans and robots [79]. In addition, the conceptual framework incorporates the situated nature of cognition through inclusion of three types of cognitive load: intrinsic, extraneous and germane. Cognitive load is based on conceptualization of the mind as being like a computer in that it processes the information it receives, rather than merely responding to stimuli [31,83,84]. Through the inclusion of work pragmatics and cognitive load, modelling of Information Gain targets for cognitive factories introduces new specificity in theory building for situated entropy.

### 6.2. Implications for Applied Research

**Figure 2:**
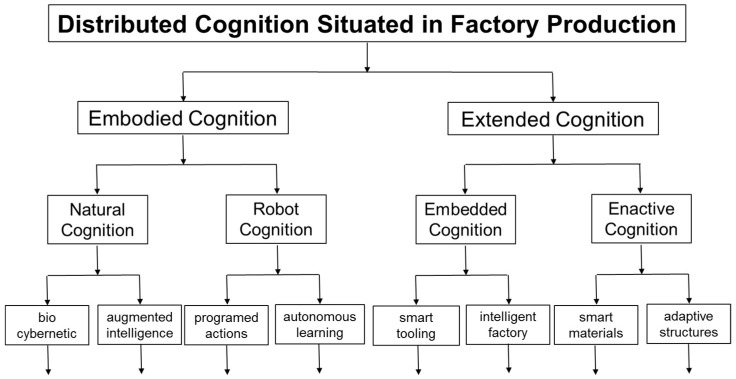
Taxonomy for distributed cognition situated in factory production.

As summarized in Figure 2, technological advances introduce new opportunities for the reduction of situated entropy: even in ETO production where there is limited scope for standardization of physical components, etc. In particular, the embodied cognition of human operatives and assembly robots can be extended by introduction of embedded cognition in smart tooling and through factory buildings. In addition, there are opportunities to bring about enactive cognition through smart materials and adaptive structures that include materials with sensor-actuator capabilities [85,86]. However, as illustrated by the example of augmented reality summarized in Table 6, it is important that Information Gain brought about by extended cognition is *Net* Information Gain. In other words, it is important to take into account and minimize the situated entropy that embedded and enactive technologies can introduce in to physical production work. In doing so, the potential introduction of technologies for extended cognition can be analysed in terms of cognitive load and work pragmatics during applied research that seeks to bring increased precision in place of vague slogans such as Industry 4.0 that are associated with cognitive factories [87]. Hitherto, there has been consideration of the net financial benefits of introducing new production technologies. However, there has been little, if any, previous consideration of Net Information Gain from the introduction of new cognitive technologies into factory work.

### 6.3. Implications for Practice

**Figure 3:**
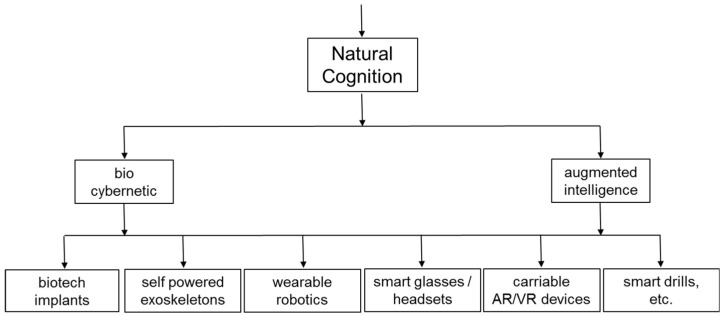
Taxonomy examples for augmenting embodied cognition of human operatives.

**Figure 4:**
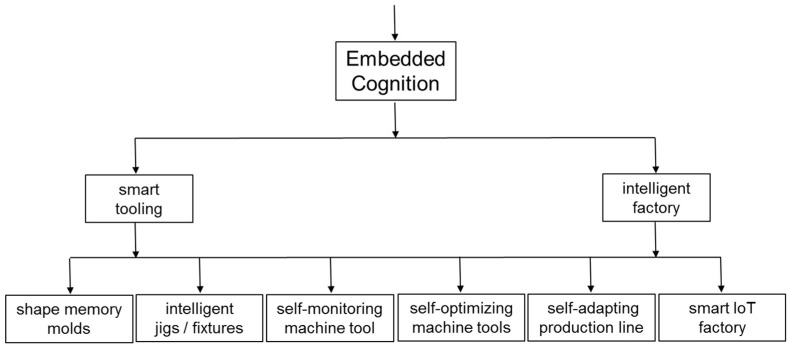
Taxonomy examples for embedded cognition.

As summarized in Figure 3 and Figure 4, practitioners are faced with an ever-increasing variety of technologies that have potential for increasing embodied cognition and extended cognition. As illustrated by Equations in Section 4 and Section 5, application of Information Theory mathematics provides a straightforward basis for analysing situated entropy and providing targets for Net Information Gain. As summarized in Table 4, Table 5, Table 6 and Table 7 this mathematics can be related easily to the practicalities of physical production in cognitive factories, such as the number of different ways of picking, orientating and placing of physical components.

Detailed measurement of production processes is essential to the management of production processes [88]. The adage, “You can’t control what you can’t measure” has its origins in the development of computational systems, and is very pertinent to the management of work processes in factories [89]. Measurement of factory work processes was formalized in the time studies and motion studies more than one hundred years ago. Time studies evolved to focus on the definition of standard times for tasks, while motion study evolved towards improving work methods. They became integrated into time and motion studies, which continue to be used within industrial engineering practices for improving work systems [90]. Building on the notion that there could be precise measurement of factory work, statistical process control was introduced in the 1930’s. This is often applied under the slogan of Six Sigma within Total Quality Management that addresses many different types of work in organizations. The expansion of measurement to many different types of work has included development of cognitive task analysis. This method involves survey research, including observation studies and face-to-face interviews, in order to determine what knowledge is being used both explicitly and implicitly during work. All of these methods involving seeing improvement as an iterative process of measurement—improvement action—remeasurement [91,92,93,94]. However, none of these involve measurement focused on situated entropy and Net Information Gain. As illustrated by the examples in Section 4, situated entropy and targets for Net Information Gain can be measured through application of Information Theory mathematics, and expressed in terms of three types of cognitive load and three types of work pragmatics. This brings a sound theoretical basis for practical measurement in the management of cognitive factories. In particular, it is important to measure situated entropy introduced by new technologies that are intended to increase Information Gain. Then, it is important to take actions to reduce situated entropy in order to ensure that the introduction of new cognitive technologies reduce rather than inadvertently increase the complexity of production systems [95,96].

## 7. Conclusions

By 2018, cognitive factories will have existed as a manufacturing systems goal for more than 10 years. Reliance on human workers for sensorimotor/psychomotor tasks in the production of individual goods continues in spite of many efforts to improve the embodied cognition of robots. Nonetheless, human operatives make many “human errors” during production work. One step towards improving the performance of both assembly robots and human peratives is modelling situated entropy. This is because situated entropy can provide a measurement of task uncertainty that is applicable to both assembly robots and human operatives. At the same time, measurement of situated entropy can provide targets for Net Information Gain.

In this paper, six contributions have been provided to advance modelling of situated entropy in cognitive factories. First, two theoretical frameworks have been brought together to structure the application of Information Theory mathematics. As summarized in Table 1, Table 2 and Table 3 these are cognitive load and work pragmatics. Second, as summarized in Table 4, Table 5 and Table 6 the conceptual framework derived from these theoretical formulations has been related to the practicalities of physical production using examples of physical components, visual control boards and assembly workstations. Third, Information Theory mathematics has been applied to the practical examples within the conceptual framework, and a preliminary methodology for modelling in practice has been introduced. In particular, five types of situated entropy have been considered: discrete equal probabilities, discrete unequal probabilities, joint entropy, conditional entropy, and differential entropy. Fourth, through relating cognitive load and work pragmatics to production expressed in terms of Information Theory mathematics, the physical artefacts of work places, such as physical components, control boards and workstations, have been reframed as carriers of Information Gain and situated entropy, which may or may not bring Net Information Gain. Fifth, as summarized in Figure 1, situated entropy has been related to four types of cognitive factories, which involve different levels of uncertainty in production operations: MTS, ATO, TTO and ETO. Sixth, the need to measure net Information Gain in the introduction of new technologies for extended cognition has been discussed in relation to a taxonomy for distributed cognition situated in factory production. Overall, modelling of situated entropy is introduced as an opportunity for improving the planning and control of cognitive factories.

## Figures and Tables

**Table 1:** Theoretical frameworks.

Theoretical Frameworks		References
Cognitive Load	Situated Cognition	[29]
	Embodied Cognition	[37]
	Embodied Cognitive Load	[30]
Work Pragmatics	Material Interaction Pragmatics	[34]
	Performative Pragmatics	[33]
	Relevance Theory	[36]

**Table 2:** Conceptual framework for modelling.

Type of Information Gain/Cognitive Load Reduction	Example Knowledge Unit	Mode of Information Gain/Cognitive Load Reduction	Design for Information Gain and Cognitive Load Reduction	
			Design In Gain Examples	Design Out Load Examples
Intrinsic	Physical components	Material Interaction Pragmatics	Design components for simplicity of assembly	End ambiguous component assembly features
Extraneous	Visual control boards	Performative Pragmatics	Design for jigs/templates for clarity and engagement	End tools stored without visual control boards
Germane	Assembly workstations	Schema Relevance	Design work cells for adaptive flexibility	End ad hoc development of factory layouts

**Table 3:** Sources of entropy.

Type of Information Gain/Load Reduction	Example of Knowledge Unit	Sources of Entropy	References
Intrinsic	Physical components	Picking Orientating Placing	[45,46]
Extraneous	Visual control boards	Conceptual Presentational Linguistic	[47,48]
Germane	Assembly workstation	Component access Work sequence Physical positioning	[49,50]

**Table 4:** Information Gain targets for one task at one workstation.

Type of Information Gain/Load Reduction	Example Knowledge Unit	Sources of Entropy	Entropy	
			Number of Different Ways of Carrying Out the Same Work	Entropy
Intrinsic	Physical components	Picking	1	0.00
		Orientation	3	1.58
		Placing	2	1.00
		Target		2.58
Extraneous	Visual control boards	Conceptual	1	0.00
		Presentational	3	1.58
		Linguistic	words not used	0.00
		Target		1.58
Germane	Assembly workstation	Component access	2	1.00
		Work sequence	2	1.00
		Physical positioning	5	2.32
		Target		4.32

**Table 5:** Targets for one task at one workstation—unintended unequal distributions.

Type of Information Gain/Load Reduction	Example Knowledge Unit	Sources of Entropy	Entropy	
			Number of Different Ways of Carrying Out the Same Work	Entropy
Intrinsic	Physical components	Picking	1	0.00
		Orientation	(2.0, 0.5, 0.5) 3	1.25
		Placing	2	1.00
		Target		2.25
Extraneous	Visual control boards	Conceptual	1	0.00
		Presentational	(1.0, 1.0, 1.0) 3	1.58
		Linguistic	words not used	0.00
		Target		1.58
Germane	Assembly workstation	Component access	2	1.00
		Work sequence	2	1.00
		Physical positioning	(4.0, 0.2, 0.2, 0.2, 0.2) 5	1.00
		Target		3.00

**Table 6:** Work instruction entropy sources for human operatives.

Type of Information Gain/Load Reduction	Example Knowledge Unit	Sources of Entropy		Entropy	
				Number of Different Ways of Carrying Out the Same Work	Entropy
Extraneous	Work Instructions	Information	Conceptual	1	0.00
			Presentation	2	1.00
			Linguistic	1	0.00
			Target		1.00

**Table 7:** AR work instruction entropy sources for human operatives.

Type of Information Gain/Load Reduction	Example Knowledge Unit	Sources of Entropy		Entropy	
				Number of Different Ways of Carrying Out the Same Work	Entropy
Extraneous	Augmented Reality Work Instructions	Information	Conceptual	1	0.00
			Presentation	1	0.00
			Linguistic	1	0.00
		Communication	Task fit	3	1.58
			Place fit	2	1.00
			Person fit	2	1.00
		Target			3.58
